# Detection of trace metallic elements in oral lichenoid contact lesions using SR-XRF, PIXE, and XAFS

**DOI:** 10.1038/srep10672

**Published:** 2015-06-18

**Authors:** Tomoko Sugiyama, Motohiro Uo, Takahiro Wada, Daisuke Omagari, Kazuo Komiyama, Serika Miyazaki, Chiya Numako, Tadahide Noguchi, Yoshinori Jinbu, Mikio Kusama, Yoshiyuki Mori

**Affiliations:** 1 Department of Dentistry, Oral and Maxillofacial Surgery, Jichi Medical University, Tochigi, Japan; 2 Advanced Biomaterials Department, Graduate School of Medical and Dental Sciences, Tokyo Medical and Dental University, Tokyo, Japan; 3 Department of Materials Engineering, Graduate School of Engineering, The University of Tokyo, Tokyo, Japan; 4 Department of Pathology, Nihon University School of Dentistry, Tokyo, Japan; 5 Graduate School of Science, Chiba University, Chiba, Japan

## Abstract

Oral lichen planus (OLP) and oral lichenoid contact lesions (OLCL) are chronic inflammatory mucocutaneous reactions with a risk of malignant transformation that alter the epithelium. OLP and OLCL have similar clinical and histopathological features and it is difficult to distinguish one from the other. Metallic restorations are suspected to generate OLCLs. Trace metal analysis of OLCL specimens may facilitate the discrimination of symptoms and identification of causative metallic restorations. The purpose of this study was to assess OLCL tissue samples for the prevalence of metallic elements derived from dental restorations, and to discriminate OLCL from OLP by using synchrotron radiation-excited X-ray fluorescence analysis (SR-XRF), particle-induced X-ray emission (PIXE), and X-ray absorption fine structure (XAFS). Typical elements of dental materials were detected in the OLCL, whereas no obvious element accumulation was detected in OLP and negative control specimens. The origin of the detected metallic elements was presumed to be dental alloys through erosion. Therefore, our findings support the feasibility of providing supporting information to distinguish OLCL from OLP by using elemental analysis.

Lichen planus was first described by Wilson in 1869. It is a common mucocutaneous lesion with an established immune-mediated pathogenesis, and often occurs orally without skin lesions^1^. Oral lichen planus (OLP) is a chronic inflammatory disease affecting stratified squamous epithelia, and it may occur anywhere in the oral cavity. The buccal mucosa, tongue, and gingiva are the most commonly affected sites. Andreasen classified OLP into six types: reticular, papular, plaque-like, erosive, atrophic, and bullous OLP^2^. The erosive, atrophic, and bullous forms are often associated with a burning sensation and cause severe pain in many cases. The histopathological features of OLP show three classic features: overlying keratinization, band-like layer of chronic inflammatory cells within the underlying connective tissue, and liquefaction degeneration of the basal cell layer^3^,^4^,^5^. The aetiology of OLP is unknown but several factors, including metal allergy, hepatitis C virus infection, endocrine disturbance, and mental stress have been implicated.

In contrast, oral lichenoid lesions (OLL) are lichen-like reactions and have clinical and histopathological features similar to those of OLP. The similarity and indices for discrimination of OLP and OLL are listed in Table 1. OLLs are often classified into the following clinical types: (1) oral lichenoid contact lesions (OLCL) induced by allergic contact stomatitis, which is commonly caused by metallic restorations; (2) oral lichenoid drug reactions (OLDR) induced by certain medications; and (3) oral lichenoid lesions in graft-versus-host disease induced by marrow grafts (OLL-GVHD)^6^,^7^. OLDR and OLL-GVHD can be distinguished on the basis of the medical history (drug treatments and marrow graft) obtained from the treatment records. However, it is difficult to distinguish OLCL from OLP because of the obscure indices for lesion location and metallic restoration. For example, some patients have developed bilateral lesions with unilateral dental restoration procedures or have developed lesions that were in the vicinity of (but not in direct contact with) the metallic restoration. Clinically, the occurrence of these conditions is considerably high, with OLP and OLCL affecting 0.5-2.2% of the population^7^. In addition, the possible risk of malignant transformation of OLP and OLCL has been documented in the literature^4^,^8^,^9^,^10^,^11^,^12^,^13^,^14^,^15^,^16^,^17^,^18^,^19^,^20^,^21^,^22^. The causative factors for OLP are unknown and steroid therapy is the only OLP treatment available. However, a definitive OLCL identification can be made based on the presence of causal metallic restorations in the vicinity^23^,^24^. Furthermore, a definitive OLCL identification can be used to prescribe removal of the causative restoration. The removal of metallic restorations is a simple procedure that does not have the side effects associated with steroid treatments commonly prescribed to treat OLCL. Furthermore, the removal of metallic restorations can cure OLCL and thereby eliminate the risk of malignant transformation, which is associated with OLCL. Therefore, to prescribe dental restoration removal as treatment, it is important to diagnose OLP and OLCL distinctively. However, the costs and physical discomfort endured by patients who have to undergo dental restoration removal should also be taken into consideration.

Patch tests are commonly used to determine metal allergy development. However, patch tests are not reliable because they often yield false positive/negative reactions or side reactions. Therefore, a better method for identifying metal allergies against dental restorations is required. The prevalence of metallic ions (eroded from the dental restorations) in the lesions could be an indicator of lesion development induced by restorations and the removal of purported restorations could be recommended. However, dental alloys are designed to resist corrosion; therefore, the concentrations of eroded and accumulated metallic ions in neighbouring mucosae can occur at levels that may be difficult to detect. In addition, biopsy specimens are obtained in small quantities and are mainly used for histopathological diagnosis. Therefore, biopsy specimens have limited availability and adequate amounts of samples are often not obtainable for analysing the distribution of metallic elements in lesions. Furthermore, the metallic element analysis requires examination of unprocessed and intact tissue specimens. Therefore, conventional elemental analysis methods are not suitable for conducting metallic elemental analyses.

The detection and chemical state analysis of eroded metallic trace elements in the soft tissues has been studied previously^25^,^26^,^27^,^28^,^29^. Synchrotron radiation X-rays were used for the analysis in these reports. The X-rays used in the analysis seldom damage the specimens and the data provide useful information. Synchrotron radiation-induced X-ray fluorescence spectroscopy (SR-XRF) provides information on elements in the specimens and X-ray absorption fine structure (XAFS) analysis provides information on the chemical state of the target element. Using these methods, trace metallic elements in biopsy specimens can be detected and analysed.

The purpose of this study was to estimate the prevalence of the trace metallic elements in OLCL and OLP by using highly sensitive and non-destructive methods such as SR-XRF, particle-induced X-ray emission (PIXE) analysis, and XAFS to estimate the feasibility to provide supporting information concerning the prevalence of dental alloy elements in the OLP and OLCL tissues to enable their discrimination.

## Results

The metallic elemental accumulation and localisation in the 15 tissue specimens were assessed by using SR-XRF and micro-PIXE. Table 2 shows the derivation, clinical diagnosis, and detected elements in the 15 specimens. The prevalence of each element was determined by using the fluorescence (characteristic) X-ray intensity of the localised area for each element. The fluorescence intensities of the accumulated metallic elements were compared with those of the surrounding tissue area (without metallic element accumulation). As shown in Table 2, significant or possible accumulations of non-intrinsic metallic elements were observed in five specimens (#7, #8, #10, #11, and #12) diagnosed as OLCL. The detected elements are the typical elements of dental materials. In contrast, no obvious accumulation was observed in the OLP specimens (#1-#6). Therefore, a difference in the prevalence of those elements between OLP and OLCL was suggested.

**Figure 1:**
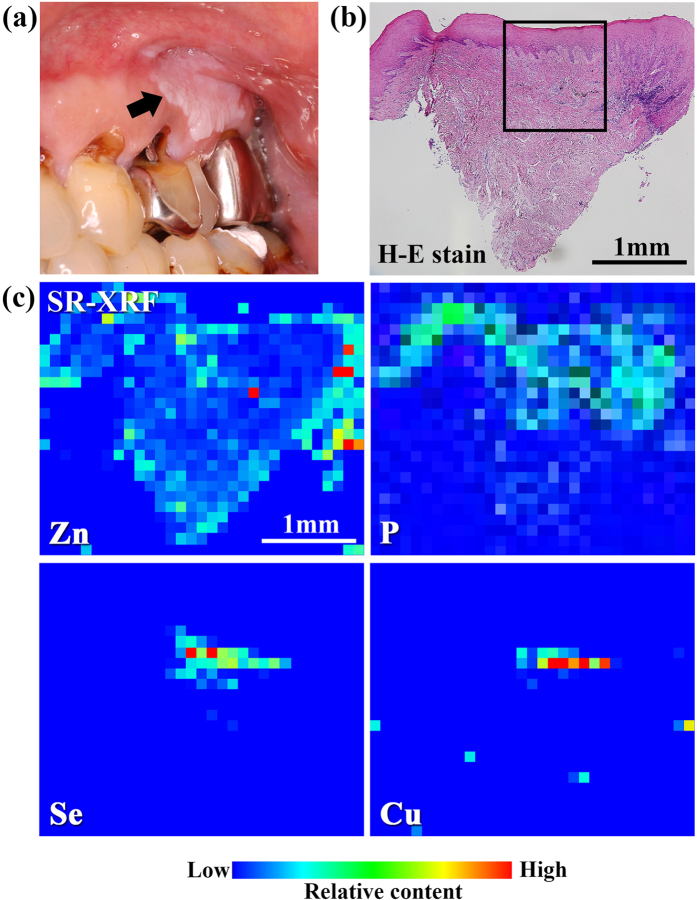
(**a**) Clinical examination, (**b**) histopathological image, and (**c**) SR-XRF elemental distribution images of specimen #7. (**a**) Hyperkeratosis was found under the tooth crown (indicated by an arrow). (**b**) Overlying keratinization, a band-like layer of inflammatory cells, and liquefaction degeneration of the basal cell layer were found. These are the characteristic features of OLP and OLCL. (**c**) Localisation of Zn, Cu, and Se in the subepithelial layer was observed. P and inflammatory cells were co-localised.

**Figure 2:**
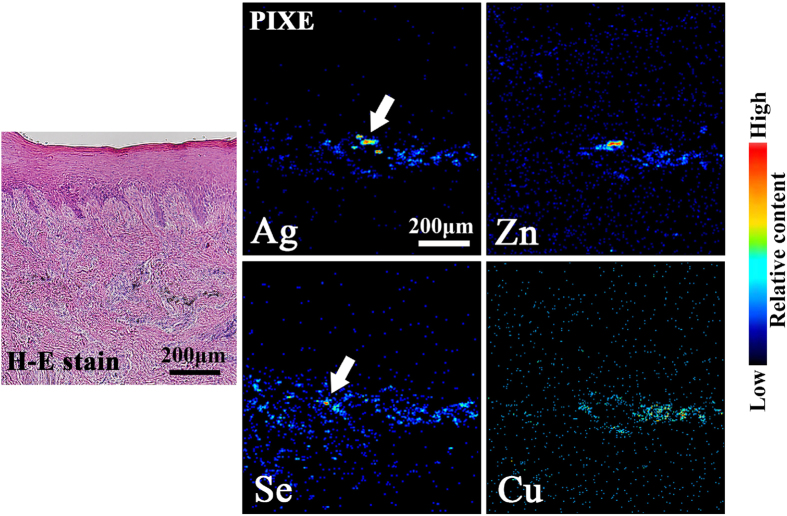
Detailed elemental distribution images obtained by using micro-PIXE of the boxed area in Fig. 1b. Ag localisation was found in the connective tissue under the epidermis. Zn, Cu, and Se also localised in the region with Ag localisation.

**Figure 3:**
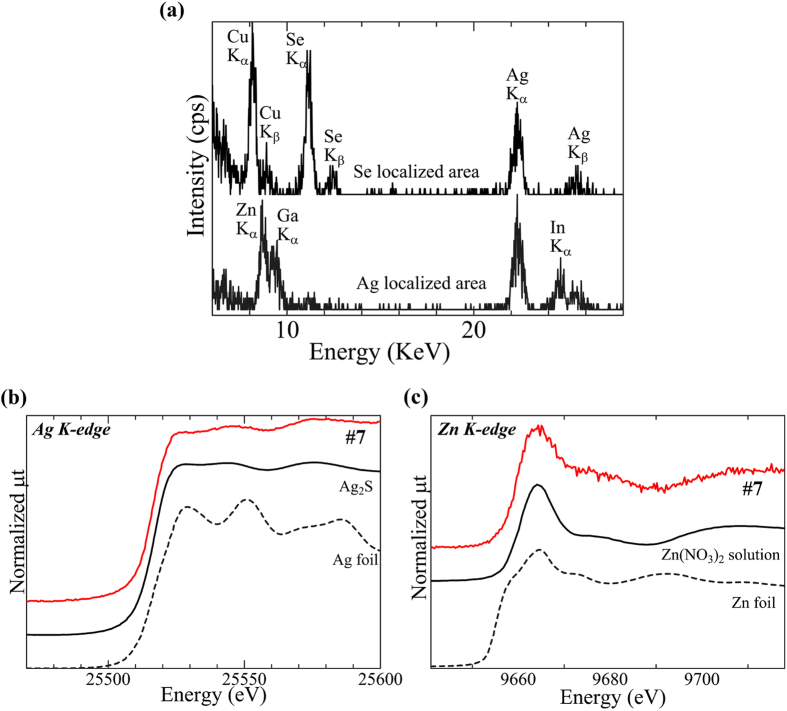
(**a**) Characteristic X-ray spectrum of the dense areas of Se and Ag, in the areas marked with white arrows in Fig. 2 (Ag and Se), and (**b**, **c**) Ag and Zn K-edge XANES spectra of specimen #7. (**a**) The peaks of Zn, In and Ga were observed in the Ag-localised area. Ag and Cu accumulated in the Se-localised area. (**b**) The Ag K-edge XANES spectrum was different from that of metallic Ag but similar to that of Ag_2_S. (**c**) The Zn K-edge XANES spectrum was different from that of metallic Zn but similar to that of Zn(NO_3_)_2_ solution. The spectral similarities indicate that Ag and Zn are not in the metallic state (metal debris). Eroded metallic restorations may be a potential source of the elements.

Figure 1 shows the clinical and histopathological examination (haematoxylin and eosin (H-E) staining), and the SR-XRF elemental distribution images of specimen #7. The specimen was obtained from an individual with hyperkeratosis under the tooth crown. The histopathological findings showed characteristic features of OLP and OLCL: overlying keratinization, band-like layer of chronic inflammatory cells within the underlying connective tissue, and liquefaction degeneration indicated by the serration of the basal cell layer. Thus, this case was diagnosed as OLCL. Localisation of Zn, Cu, and Se was observed in the sub-epithelial layer. P was located in the inner epithelium layer and was co-localised in the region with inflammatory cells. The elemental distribution in the rectangular area shown in Fig. 1 was analysed using micro-PIXE as shown in Fig. 2. Micro-PIXE was used to visualize localisation of Ag, Zn, Cu and Se. The characteristic X-ray spectra of Ag and Se high-density regions (arrows) are shown in Fig. 3(a). In the Ag-localised region, peaks identified as Zn, In, and Ga were also observed. A commercially available silver alloy produced by Casting Silver S (GC Corp., Tokyo, Japan) has the composition of Ag – 24 In – 2.5 Zn – 2 Ga – 1 Pd (wt%). Ag, In, and Ga are the alloy components of dental silver alloy and are not endogenously present in the body. Therefore, the presence of these elements in lesions indicates that they most likely originated from dental alloys. The Ag K-edge XANES spectrum was different from the metallic Ag spectrum and similar to the Ag_2_S spectrum (Fig. 3b). The XANES spectrum of Zn was also different from that of metallic Zn and similar to that of Zn(NO_3_)_2_ solution (Fig. 3c). These results indicate that Ag and Zn were derived from the dissolution of dental alloys and had accumulated in the mucosal tissue. Ag has considerably low solubility in aqueous solution; therefore, dissolved Ag precipitates as Ag_2_S, which is chemically stable. In contrast, the chemical state of Zn in this specimen was similar to that of Zn in aqueous solution. Given the high solubility of Zn in aqueous solution, it may have accumulated in the tissue as aqueous Zn.

Zn and Cu are essential trace elements for humans; therefore, it is difficult to distinguish the accumulated elements of eroded dental alloy components from pre-existing endogenous trace elements. However, the excess levels of Zn and Cu in a restricted region and the chemical state information suggest that these elements were most likely derived from dental alloys.

As shown in Figs. 1 and 2, Ag, Zn, and Cu were localised in a vicinal small region, whereas Se was widely distributed around those elements. The characteristic X-ray spectrum of Se in a localised region (Fig. 3a) showed that Ag and Cu also accumulated along with Se. Se is not a component of dental alloys or dental material but is an essential element for humans. Therefore, the detected Se was considered to be present naturally in the human body.

**Figure 4:**
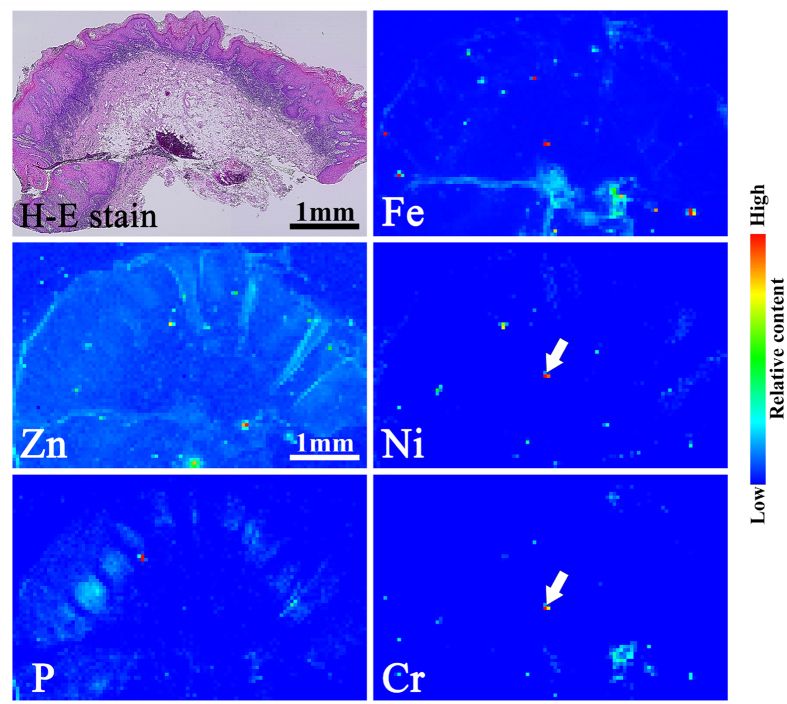
Histopathological image of haematoxylin-eosin stained (H-E stained) specimen #12 and SR-XRF elemental distribution images of specimen #12. Clear localisation of Fe, Cr and Ni was observed. The localisation of P mainly corresponded to the layer of infiltrating inflammatory cells.

**Figure 5:**
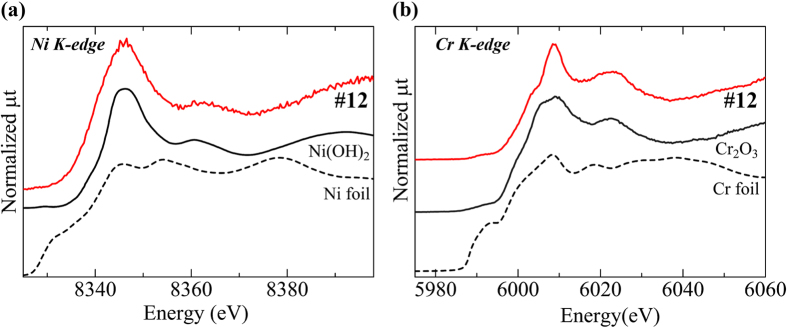
Ni and Cr K-edge XANES spectra of the area in specimen #12 indicated with a white arrow in Fig. 5. (**a**) The Ni K-edge XANES spectrum was different from that of metallic Ni but similar to that of Ni(OH)_2_. (**b**) The Cr K-edge XANES spectrum was different from that of metallic Cr but similar to that of Cr_2_O_3_.

Figure 4 shows the histopathology and elemental distribution images of specimen #12, which was obtained from the buccal mucosa. The histopathological findings showed the characteristic features of keratotic lesions. Remarkable localisation of Fe, Cr, and Ni was observed. These elements are the major components of stainless steel. The XANES spectra data (Fig. 5) indicate that Ni and Cr were not present in the metallic state but had similarity to Ni(OH)_2_ and Cr_2_O_3_. As with specimen #7, Ni and Cr in specimen #12 were assumed to be derived from the dissolution of stainless steel and accumulated in an aqueous state (Ni) and as an oxide (Cr).

**Figure 6:**
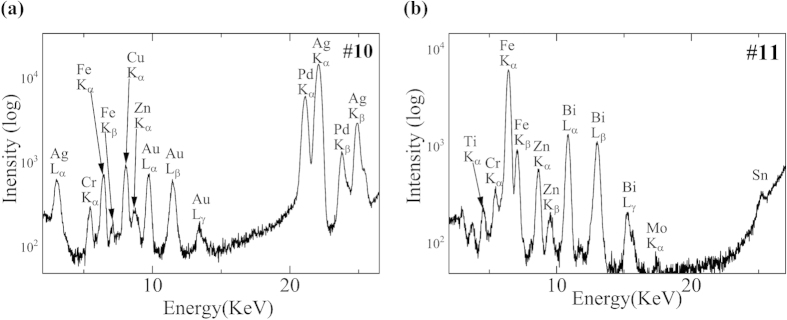
SR-XRF spectra of specimens #10 and #11. (**a**) The peaks of Ag, Pd, Au, and Cu were observed. These are the major components of the most popular dental alloy (Au-Pd-Ag-Cu alloy). (**b**) The peak of Bi was observed. Bi is a dental material contained in root canal sealers.

Figure 6(a) shows the XRF spectrum of specimen #10, which shows the accumulation of Ag, Pd, Au, and Cu. These are the major components of Au-Ag-Pd-Cu alloy, which is the most popular dental alloy in Japan. Figure 6(b) shows the accumulation of Bi and Zn (Fe originates from the blood) in specimen #11. Bi is not an alloy component but is present in root canal sealers as bismuth subcarbonate with zinc oxide. Specimen #8 shows localisation of Ag, Ni, Cu, and Zn. Thus, five of six specimens that were clinically and histopathologically diagnosed as OLCL showed obvious accumulation and localisation of dental alloy-derived elements.

**Figure 7:**
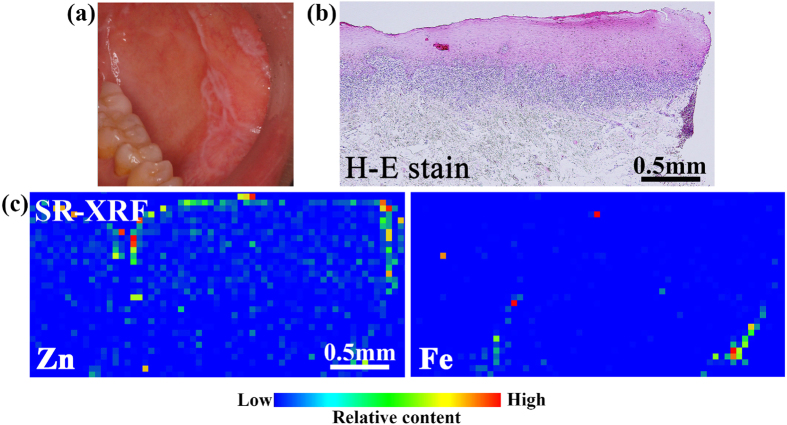
(**a**) Clinical examination, (**b**) histopathological image of H-E stained specimen #4, and (**c**) SR-XRF elemental distribution images of the same region used for histopathological observation. (**a**) Hyperkeratosis was found on the buccal mucosa, but no metallic restorations were found in the vicinity. (**b**) Overlying keratinization, a band-like layer of inflammatory cells and liquefaction degeneration of the basal cell layer were assessed. These features were similar to the OLCL (Fig. 1b). (**c**)There was no remarkable accumulation of metallic elements. Zn and Fe were distributed widely and not localised.

The clinical examination data (Fig. 7a) and histopathological examination data (Fig. 7b) for specimen #4 are shown in Fig. 7. Hyperkeratosis was found on the bilateral buccal mucosa; however, metallic restorations were not present. Histopathology showed that the three characteristic features, which are similar to those of OLCL (Fig. 1b), were present. This case was diagnosed as OLP based on clinical and histopathological findings. There was no remarkable accumulation of dental alloy elements or essential elements such as Zn and Fe. These elements were prevalent throughout the specimen and were not localised. In addition, the intensity of the fluorescent X-ray of these elements in specimen #4 was lower than that of the OLCL specimens. Other OLP specimens and the negative control (normal mucosal tissue) showed no characteristic accumulation of metallic elements. Therefore, accumulation of dental alloy elements was not detected in OLP specimens.

## Discussion

Distinguishing OLCL from OLP is useful for determining the treatment plan. However, OLP and OLCL have similar clinical and pathological features, which makes their distinction difficult. Eroded metallic restorations that are in direct contact or in the vicinity of OLCL have been associated with OLCL pathogenesis^24^. Therefore, the prevalence of dental alloy elements in OLCL and OLP tissue could be used for distinguishing OLCL from OLP. Studies to date have not been able to accurately detect dental alloy elements because of the various limitations associated with methods used for trace element analysis in the tissue specimens.

OLP and OLCL are not tumoral diseases; therefore, resections of entire lesions are not usually applied and resected specimens that represent a small portion of the lesions (a size of several millimetres is typical) are obtained with the aim of histopathological diagnosis. All biopsy specimens are formalin-fixed and paraffin-embedded as part of the standard processing method used for histopathological diagnosis. Therefore, because of the limited availability of samples, most of the tissue samples used in this study were paraffin-embedded specimens. There were additional advantages of developing a methodology that uses paraffin-embedded specimens. Because the paraffin-embedded specimens of all patients are usually stored for future use (such as additional diagnostic tests), previously collected and stored samples could also be used for detecting dental elements in the specimens. Furthermore, elemental analysis of the paraffin-embedded specimens can be performed using a small amount of sample and without destroying the sample. Either inductively coupled plasma atomic emission spectroscopy (ICP-AES) or mass spectroscopy (ICP-MS) is generally used for trace elemental analysis. Although these methods have high sensitivity, they require a larger quantity of specimens and the specimens cannot be reused for other analyses. Therefore, these methods were not used for the trace elemental analysis of OLP and OLCL specimens.

SR-XRF and micro-PIXE are highly sensitive methods for the detection of trace elements without destroying the sample, and they can detect the elements in ordinary paraffin-embedded specimen slices. Micro-focused SR-XRF was used to obtain elemental distribution images with two-dimensional mechanical scanning of the specimens. The scanning area of SR-XRF is wide with high sensitivity, therefore, it is suitable for the measurement of entire OLP and OLCL specimens. The focal size of the capillary focused X-ray (approximately 20 μm in KEK-PF BL-4A) is particularly appropriate for this purpose. However, the detailed distribution at micrometre resolution could not be provided by this method. Additionally, in some SR-XRF facilities the energy of the incident X-ray is limited and insufficient for the excitation of heavy elements such as Ag, Pd, In, and Sn, which are the major elements of the dental alloys. However, Micro-PIXE can provide detailed elemental distributions for most elements. Therefore, through the complementary usage of these two methods, the detailed distribution of dental alloy elements in mucosal specimens can be efficiently provided. By comparing the elemental distribution images with histopathological images, the effect of the accumulated foreign object-derived elements on the surrounding tissue can be revealed.

In the present study, the typical elements of dental materials were detected in five of six specimens diagnosed as OLCL, whereas no obvious elemental localisation was observed in the six OLP specimens and in three negative control specimens. Typical dental alloy elements, such as Ag, In, Au, and Pd, which are not normally present in the body, were detected in OLCL specimens and were likely to be of dental alloy origin. However, other elements, including Zn, Cu, Ni, and Cr, are major components of dental alloys and are also essential trace elements found in humans. Therefore, their derivation could not be determined definitively. However, there was a remarkable localisation of the elements, as shown in Fig. 5, and foreign origin could thus be considered as a possible candidate source for the elements. Therefore, accumulation of metallic elements related to the dental alloys in OLCL was assumed. As noted above, to discriminate between the elements of the dental alloys and the essential trace elements, quantitative evaluation is necessary. An approximate estimation of the concentration of these elements was attempted using trial calibration standards. The results are shown in Table S3 in the supporting information. Under these conditions, the concentrations of the trace essential elements in the negative control specimen were similar to the reported concentrations in the normal buccal mucosa, whereas the concentrations in OLCL were higher than those of normal mucosa. Therefore, progress in the quantitative estimation of these elements in mucosal tissues is necessary for accurate discrimination of OLP and OLCL.

Dental alloy debris is often generated from the cutting and polishing of metal restorations during dental treatments. The debris occasionally penetrates into the mucosa. Additionally, contamination from the microtome blade may occur during the preparation of thin sections^28^. Incorporated metal debris can act as an irritant and induce local inflammation, but not sufficiently to cause OLCL. Therefore, metal debris should not be considered a major source for the accumulated elements in the lesions. XAFS analysis provides information on the chemical state of localised elements and can discriminate among alloys, dissolved ions, and other chemical species. Therefore, debris contamination could be easily excluded from the suspected elemental localization by XAFS analysis. The detected elements in the OLCL specimens did not occur in the metallic state and were estimated as aqueous ions (Ni, Zn), sulphide (Ag), and oxide (Cr). Ag and Cr have low solubility and therefore form precipitates to attain a stable chemical state in body fluids. Taken together, the data indicate that the accumulated metallic elements were most likely derived from the erosion of dental metallic restoratives and may have influenced OLCL development. Thus, the combined use of elemental distribution analysis (SR-XRF and micro-PIXE) and XAFS can be applied to obtain useful information regarding the incorporation of metallic elements in mucosa and could be indicative of the causative or related material involved in OLCL development.

To estimate the *in vivo* erosion of the dental alloys, the chemical state of the eroded and accumulated metallic elements in the tissues surrounding the implanted dental alloys was previously investigated^30^. In that study, various dental alloys were implanted subcutaneously in mice, and the dried surrounding tissues were subjected to XAFS analysis without formalin fixation and paraffin embedding. Ni ions, Cr_2_O_3_, and Ag_2_S were identified in the tissues surrounding nickel-chromium, cobalt-chromium, and silver alloy. The chemical state of the detected elements was similar that of the elements in the present study that were derived from the erosion of dental metallic restorations.

In the present study, specimens were formalin-fixed and paraffin-embedded. Thus, their preparation process may have affected the elemental distribution and chemical state were concerned. However, concerning the chemical state, the similarity of the chemical state in the present study and that of specimens that were simply dried^30^ suggests that the effect of the fixation and embedding process on the chemical state of the accumulated metallic elements would be minimal. Hart *et al.*^31^ reported that the elemental distribution of eroded alloy elements into tissues from the Co-Cr hip joint was similar between the formalin-fixed and the fresh-frozen specimens. Therefore, the effect of the fixation and embedding process on the distribution and chemical state would be acceptably small. However, the loss of trace elements, especially dissolved elements in the ionic state, from the tissue during the fixation and embedding process is still a concern. In such cases, the frozen-sections should be used.

In the OLCL specimens (#7), Se was detected within the same region as Ag and Cu as shown in Fig. 2 and Fig. 3. Se is not a component of dental alloys or dental material, but is an essential element in humans. Therefore, the detected Se was considered to be endogenously present. One of the present authors has already reported the relationship among Se, Ag, and Hg in oral mucosa^26^. Ag, Cu, and Hg have high affinity for chalcogen atoms such as S and Se. The interactions among Ag, Cu, Hg, and Se are suggestive in such cases. Some types of selenoproteins known as the selenium complex are present in the human body. A comparative analysis of the elemental distribution data and the data obtained from H-E stained images in Fig. 1 showed that Ag, Cu, and Se were not localised in the region of inflammatory cell infiltration; rather, they were near the post-capillary venule. This finding indicates that selenoprotein is supplied from the circulation. Therefore, the reaction between foreign-origin Ag and Cu and intrinsic Se may occur through the vasculature.

In the present study, the clinical and histopathological diagnoses of OLP and OLCL were performed by a highly skilled oral pathologists who are specialists in the discrimination of OLP and OLCL. Therefore, the diagnosis and the elemental analysis results were in agreement. However, in most medical facilities, OLP/OLCL discrimination is not performed thoroughly. Therefore, additional information is required to discriminate between the two disease conditions. At present, various metallic materials and devices are widely used in the dental and medical fields. The safety of these materials is determined using strict corrosion tests. However, the data obtained in this study indicated that some accumulation of metallic elements, which was suspected to be derived from dental alloys, can occur. In particular, the accumulation of non-intrinsic dental alloy elements, e.g., Ag, Pd, In, and Sn, was suggested to be derived from metallic dental restorations. The derivation estimation was difficult for the accumulation of intrinsic elements such as Cr, Fe, Cu, and Zn. However, the remarkable localisation of these elements suggested the role of an extrinsic factor such as derivation from dental alloys. A relationship between OLCL and contacted metal restorations has been suggested^23^,^24^. Currently, differentiation of OLCL and OLP is based only on clinical and histopathological findings, but there is no clear evidence concerning the effect of adjacent metallic restorations on the diagnosis of OLCL. Therefore, OLCL and OLP are symptomatically treated with steroids in the same way, even though they have different etiologies. There is also a risk of malignant transformation. If OLCL is caused by dental restoration, then radical treatment with removal of the causative restoration is desirable. Identification of the causative restoration by using the methods proposed in this study may enable such radical treatment. The accumulation of metallic elements observed in this study suggests the feasibility of providing supporting information to diagnose OLCL.

In the present study, the feasibility of trace metallic elemental analysis performed using SR-XRF, micro-PIXE, and XAFS analyses for detection of accumulated metallic elements from dental restorations in the mucosal tissue of OLCL was examined, and the data indicated that:

1. Typical elements of dental materials were detected in five of six specimens diagnosed as OLCL, whereas no obvious elemental localisation was observed in six OLP specimens and three negative control specimens.
2. With XAFS analysis, the detected elements in OLCL specimens were not in the metallic state. This finding indicates that the accumulated elements were not derived from contaminating metals; rather, they were most likely derived from the eroded metallic restorations.
3. It is feasible to provide supporting information to distinguish OLCL from OLP with complementary usage of SR-XRF, micro-PIXE, and XAFS.

In future studies, the consistency of the accumulated metallic elements and the adjacent dental alloy components should be assessed. In addition, the threshold of the accumulated elemental concentrations and number of specimens for diagnosis of metal-induced OLCL should be estimated using a quantitative analysis. Additional information on elemental concentrations in the oral mucosa and metal restorations would provide better indices for the differential diagnosis of OLP and OLCL.

## Methods

### Specimens

Oral mucosal tissues were supplied by the Dental Hospital of Nihon University School of Dentistry, Tokyo, Japan and Jichi Medical University Hospital, Tochigi, Japan. The samples included 15 residual biopsy materials embedded in paraffin, including six OLP samples and six OLCL samples with established clinical and histopathological diagnoses. All patients provided written informed consent and the study protocol was approved by the Ethical Committees of Nihon University (2012–14) and Jichi Medical University Hospital (A12–27). The methods were conducted in accordance with the approved guidelines; all samples were anonymous and given the original numbers on the date of the study. OLP and OLCL were diagnosed by a highly skilled oral pathologist. The OLP/OLCL diagnosis was based on the presence of a contact reaction to metallic dental restorations. In addition, three samples that were obtained from the safety margin of a resected squamous cell carcinoma specimen from the tongue, the bucca, and the gum were prepared as a control. Adjacent sections of each sample with a thickness of 8 μm were placed on Kapton^®^ film (12.5 μm thickness; Du Pont-Tray Co., Ltd, Tokyo, Japan) and subjected to elemental analysis. The elemental distribution data, including relative content, distribution, and chemical state of the element, were analysed in conjunction with the histopathology data.

### SR-XRF analysis

XRF analyses were conducted at BL-4A of the Photon Factory in the High Energy Accelerator Research Organization (KEK-PF, Tsukuba, Japan) and BL37XU of the SPring-8 (Hyogo, Japan). The energy of the incident X-ray was 12.9 KeV (BL-4A) and 30 KeV (BL37XU). The incident X-ray was micro-focused in both facilities for the micro-area analysis. The X-ray was focused into 100 μm using poly-capillary optics at BL-4A and into 2 × 4 μm using Kirkpatrick-Baez mirror optics at BL37XU. The specimens were irradiated by the micro-focused X-ray, and the specimen stage was scanned in the X-Y directions, two-dimensionally, to obtain the areal elemental distribution images. The size of the scanned areas varied within several millimetres, and the scanning steps varied from 5 μm to 50 μm. The obtained XRF spectra in BL-4A were processed using PyMca software (Version 4.7.3). In addition, the XRF spectra were measured for 300 sec at the spots containing metallic elements.

### Micro-PIXE analysis

To obtain high-resolution elemental distribution images, some specimens were also used for micro-PIXE analysis. Micro-PIXE analyses were conducted at the National Institute of Radiological Sciences (Chiba, Japan). An accelerated and micro-focused proton beam (3.0 MeV, 2 μm beam diameter) with raster scanning was applied over the target area of the specimen (maximum area of 2 × 2 mm). The generated characteristic X-rays were collected using a Si(Li) and CdTe detector to obtain elemental distribution images and characteristic X-ray spectra. The obtained data were processed with OMDAQ2007 software (Version 1.3.71.669), and the elemental distribution images and the characteristic X-ray spectra of the region of interest were obtained.

### XAFS analysis

For the typically accumulated metallic elements, XAFS analyses were conducted to reveal the chemical state of the accumulated metallic elements in the mucosal tissues. XAFS analyses were conducted at BL-4A and NW-10A of the KEK-PF. The X-ray absorption near the edge structure (XANES) spectra of the target elements (e.g., Ag, Ni, and Zn) was measured with the fluorescent XAFS method using a multi-element solid-state detector (Canberra, Connecticut, USA). Reagent-grade Ag_2_S, Cr_2_O_3_, and Ni(OH)_2_ were used as the standards for XAFS analysis. The XANES spectrum of Zn(NO_3_)_2_ solution was measured according to methods described by Numako *et al.*^32^.

## Additional Information

**How to cite this article**: Sugiyama, T. *et al.* Detection of trace metallic elements in oral lichenoid contact lesions using SR-XRF, PIXE, and XAFS. *Sci. Rep.* **5**, 10672; doi: 10.1038/srep10672 (2015).

## Supplementary Material

Supplementary Information

## Figures and Tables

**Table 1:**
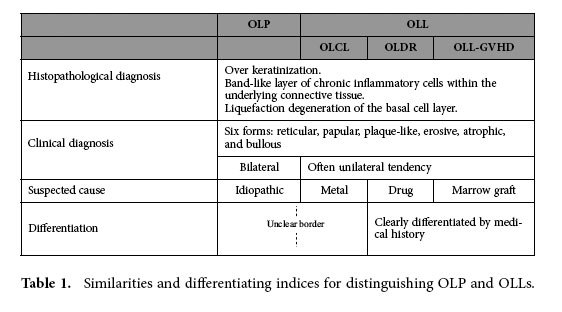
Similarities and differentiating indices for distinguishing OLP and OLLs.

**Table 2:**
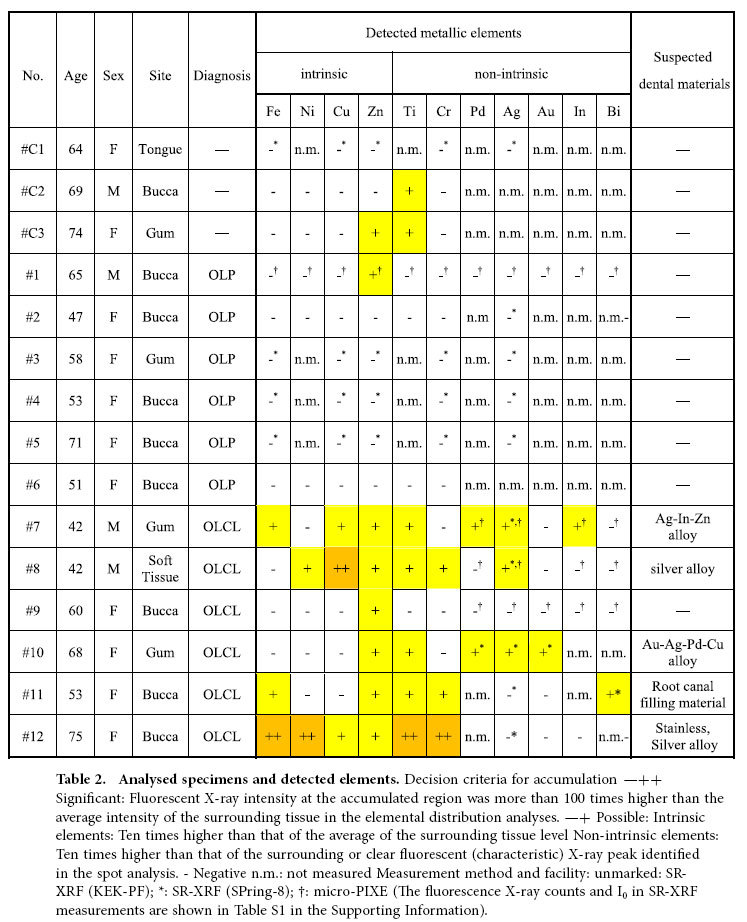
Analysed specimens and detected elements.
