# Supplementary material for: Detection of trace metallic elements in oral lichenoid contact lesions using SR-XRF, PIXE, and XAFS
Source: Sci Rep. 2015 Jun 18;5:10672. doi: 10.1038/srep10672 (PMC4471725; doi:10.1038/srep10672)
Supplement: Supplementary Information [file srep10672-s1.pdf]

Supporting Information

Detection of trace metallic elements in oral lichenoid contact lesions using SR-XRF, PIXE, and XAFS

*Tomoko Sugiyama, Motohiro Uo**, *Takahiro Wada, Daisuke Omagari, Kazuo Komiyama, Serika Miyazaki, Chiya Numako, Tadahide Noguchi, Yoshinori Jinbu, Mikio Kusama, Yoshiyuki Mori*

Table S1 Fluorescence X-ray counts (cps) of each element shown in Table 2 and the detailed measurement conditions

No.	Diagnosis		detected metallic elements											I_0		
			intrinsic				non-intrinsic							**KEK-PF (a)**	**KEK-PF (b)**	**SPring-8**
			**Fe**	**Ni**	**Cu**	**Zn**	**Ti**	**Cr**	**Pd**	**Ag**	**Au**	**In**	**Bi**			
**#C1**	Control	Max	77	n.m.	12	46	n.m.	32	n.m.	20	n.m.	n.m.	n.m.	—	—	140174.4
		Average	36.94±6.67		4.65±2.18	5.16±3.55		16.82±4.03		9.54±3.07						
**#C2**	Control	Max	524	35	72	636	266	26	n.m.	n.m.	n.m.	n.m.	n.m.	—	3075.1	—
		Average	58.21±30.97	10.59±3.36	28.73±6.17	107.00±64.44	11.08±4.75	9.91±3.11								
**#C3**	Control	Max	381	48	53	908	201	30	n.m.	n.m.	n.m.	n.m.	n.m.	—	3479.3	—
		Average	72.75±41.20	12.26±3.01	30.96±4.79	41.51±7.84	13.69±8.62	12.23±2.94								
**#1**	OLP	Max	μ-PIXE	μ-PIXE	μ-PIXE	μ-PIXE	μ-PIXE	μ-PIXE	μ-PIXE	μ-PIXE	μ-PIXE	μ-PIXE	μ-PIXE	—	—	—
		Average														
**#2**	OLP	Max	230	49	88	1253	107	36	n.m.	12	n.m.	n.m.	n.m.	—	3395.3	140334.0
		Average	98.96±.11.99	18.87±2.72	75.46±4.23	256.00±67.80	24.29±2.82	17.86±1.99		3.19±1.82						
**#3**	OLP	Max	218	n.m.	15	37	n.m.	31	n.m.	42	n.m.	n.m.	n.m.	—	—	140324.1
		Average	35.95±6.87		4.61±2.21	4.56±2.46		16.61±4.17		9.62±3.19						
**#4**	OLP	Max	62	n.m.	25	35	n.m.	34	n.m.	26	n.m.	n.m.	n.m.	—	—	133888.3
		Average	35.55±5.97		4.21±2.09	3.75±2.16		16.45±4.08		9.00±2.99						
**#5**	OLP	Max	45	n.m.	12	24	n.m.	25	n.m.	20	n.m.	n.m.	n.m.	—	—	145385.2
		Average	28.46±4.63		3.8±1.75	4.55±2.22		13.04±3.07		7.67±2.44						
**#6**	OLP	Max	1650	68	284	1612	148	170	n.m.	n.m.	n.m.	n.m.	n.m.	—	3261.5	—
		Average	189.03±86.33	25.99±4.34	107.89±12.78	356.02±26.57	35.33±10.00	24.55±9.78								
**#7**	OLCL	Max	440	9	385	2052	101	18	μ-PIXE	μ-PIXE	μ-PIXE	μ-PIXE	μ-PIXE	3092.8	—	—
		Average	17.76±13.8	1.52±0.57	6.75±6.00	24.15±16.32	1.99±1.59	1.99±0.44								
**#8**	OLCL	Max	66	52	512	1104	53	63	μ-PIXE	μ-PIXE	μ-PIXE	μ-PIXE	μ-PIXE	2835.5	—	—
		Average	7.68±3.42	1.03±0.42	2.71±1.32	12.34±5.90	1.43±0.89	1.41±0.75								
**#9**	OLCL	Max	522	9	76	1005	5	27	μ-PIXE	μ-PIXE	μ-PIXE	μ-PIXE	μ-PIXE	95768.4	—	—
		Average	67.81±4.98	5.30±0.99	59.95±3.71	18.07±5.40	2.15±0.69	19.86±1.89								
**#10**	OLCL	Max	162	11	51	279	48	27	+ ‡	594	+ ‡	n.m.	n.m.	9616.6	—	141630.0
		Average	66.92±10.95	4.45±1.53	36.84±4.69	15.71±6.87	2.43±1.49	17.64±3.27		16.09±9.71						
**#11**	OLCL	Max	2412	13	53	3161	96	140	n.m.	33	- ‡	n.m.	+ ‡	6187.7	—	147890.0
		Average	42.76±7.96	3.89±1.12	40.46±3.06	16.43±7.32	1.48±1.09	11.96±2.45		13.46±4.26						
**#12**	OLCL	Max	27883	2082	3160	13783	5591	3922	n.m.	24	- ‡	- ‡	n.m.	—	2977.1	145361.2
		Average	189.48±253.50	18.32±2.95	72.44±62.94	235.40±45.50	24.74±13.12	19.77±57.98		9.60±3.23						

+ ‡: fluorescent/characteristic X-ray peak was identified in the spot analysis at SPring-8.
- ‡: fluorescent/characteristic X-ray peak was not detected at SPring-8.

Detailed measurement conditions;

KEK-PF, BL-4A Distance between specimen and detector : 35 mm
Detector: (a) Si(Li) detector (PGT Inc., 30 mm^2)
(b) Silicon multi-cathode detector (Voltex-EX, Seiko EG&G)

SPring-8, BL-37XU Distance between specimen and detector: 300 mm

To roughly estimate the metallic elements contained in the mucosal specimens, trial standard specimens for the concentration calibration were prepared. Acetylacetonate mixed with the elements (500 ppm for Cr, Ni and Zn; 40 ppm for Cu) was then mixed with monomers consisting of bisphenol A-glycidyl methacrylate and triethylene glycol dimethacrylate. The mixed monomers were polymerized to form a thin film shape and subjected to SR-XRF measurement at BL-4A in KEK-PF under the same measurement conditions[S1]. The fluorescent X-ray intensity of the standard specimens was normalized by the film thickness as shown in Table S2.

Table S2 Fluorescence X-ray intensity of film-shaped concentration standards

	Ni	Cu	Zn	Cr
Concentration (ppm)	497	41	497	502
thickness (μm)	134	82	133	169
fluorescence X-ray intensity (cps)	8596	812	12603	5545
normalized intensity (cps/μm)	64	10	95	33

The concentrations of the accumulated elements were roughly estimated for three specimens (#C2 for the control, #2 for OLP, #12 for OLCL) and presented in Table S3 using the above-described calibration standards. The concentrations were estimated taking into consideration that the tissue specimen was sectioned at a thickness of 8 μm in thickness.

The elemental concentrations of sound buccal mucosa were previously estimated by PIXE and reported by Ishibashi et al.[S2] as follows (ppm in dry weight);

Ni: 7.86±21.23, Cu: 18.93±26.34, Zn: 66.19±30.41, Cr: 4.65±6.85

The estimated concentrations in Table S3 in the control specimens were similar to the above-reported values, whereas the concentration in OLCL was increased.

Table S3 Estimated concentration (ppm) of Cr, Ni, Cu, and Zn in control, OLP, and OLCL

		Ni	Cu	Zn	Cr
Control #C2	Max	34.00	37.00	420.00	50.00
	Average	10±3.3	15±3.1	70±42	19±6.0
OLP #2	Max	240.00	380.00	>> 1000	350.00
	Average	91±13	200±11	840±220	170±19
OLCL #12	Max	>> 1000	>> 1000	>> 1000	>> 1000
	Average	89±14	190±160	770±150	190±550

References

[S1] Sugiyama, T., Uo, M., Wada, T. & Mori, Y. submitted for publication in *J. Synchorotron Rad.*

[S2] Ishibashi, S., Sugiyama, Y., Nakamura, M. & Sekiyama, S. (2003). The trace element analysis of normal oral mucosa. *Dent. J. Iwate Med. Univ.*, **28**, 76-84.
